# High Rate of Oral *Candida* Detection in Dependent Japanese Older People

**DOI:** 10.3390/geriatrics5010021

**Published:** 2020-03-24

**Authors:** Mari Matsumura, Hideo Shigeishi, Cheng-Yih Su, Rumi Nishimura, Kouji Ohta, Masaru Sugiyama

**Affiliations:** 1 Department of Public Oral Health, Program of Oral Health Sciences, Graduate School of Biomedical and Health Sciences, Hiroshima University, Hiroshima 734-8553, Japan; m183935@hiroshima-u.ac.jp (M.M.); otkouji@hiroshima-u.ac.jp (K.O.); masaru@hiroshima-u.ac.jp (M.S.); 2 Department of Oral Health Management, Program of Oral Health Sciences, Graduate School of Biomedical and Health Sciences, Hiroshima University, Hiroshima 734-8553, Japan; d181880@hiroshima-u.ac.jp; 3 Department of Oral Epidemiology, Program of Oral Health Sciences, Graduate School of Biomedical and Health Sciences, Hiroshima University, Hiroshima 734-8553, Japan; r-nishimura@hiroshima-u.ac.jp

**Keywords:** *Candida albicans*, *Candida glabrata*, *Candida tropicalis*, dependent older people, oral moisture, denture

## Abstract

The aim of this study was to clarify the association between oral *Candida* detection and the dependency status of older people. This study included 31 older people aged ≥70 years who had a certified need for long-term care or support and received care in a local day care center; it also included 27 independent older patients aged ≥70 years who visited Hiroshima University Hospital. Oral *Candida* was detected by a polymerase chain reaction using swab samples from the tongue surface. Oral moisture was significantly reduced in dependent older people, compared with independent older people (*p* = 0.003). There was a weak negative relationship between numbers of bacteria and levels of oral moisture (Spearman’s rank correlation coefficient; R = −0.29, *p* = 0.01). Dependent older people exhibited a significantly higher rate of *Candida albicans* detection (35.5%) compared with independent older people (11.1%). Dependent older people also exhibited a higher rate of concurrent detection of both *C. albicans* and *Candida glabrata* (32.3%) compared with independent older people (11.1%), but this difference was not statistically significant. Thus, dependent older people may be more susceptible to oral *C. albicans* infection, compared with independent older people. Good oral hygiene is necessary to prevent oral *Candida* infection in dependent older people.

## 1. Introduction

The oral cavity contains diverse microbial communities, consisting of bacteria, fungi, and viruses. Recent research has shown that oral diseases (e.g., periodontitis) are associated with high serum HbA1c levels and low serum vitamin C levels, suggesting an important relationship between oral health and general health condition [1,2]. Therefore, maintenance of good oral hygiene is vital for ensuring oral and general health at all stages of life. Notably, poor oral hygiene is associated with risks of aspiration pneumonia and oral candidiasis in older people [3,4]. Aspiration pneumonia occurs due to swallowing difficulties (i.e., dysphagia) and the aspiration of oral resident bacteria in older people. In addition, oral candidiasis is a common fungal infection in older people [5]. Oral candidiasis is caused by an impaired immune mechanism, medication use, xerostomia, and denture use in older people [5,6]. Common clinical features of chronic atrophic candidiasis (i.e., denture-related stomatitis due to the presence of *Candida*) are erythema and edema of the mucosa. The long-term use of dentures and poor oral hygiene presumably contributes to the risk of oral candidiasis by producing an environment ideal for growth of *Candida*. In addition, decline in activities of daily living may be associated with oral candidiasis due to poor oral health care in older people.

Thus far, the prevalence of oral *Candida* in older Japanese people has not been fully investigated. The aim of this study was to clarify the association between oral *Candida* detection and the dependency status of older people. Therefore, the present study investigated the rate of *Candida* detection in the oral cavity in dependent older people who had a certified need for long-term care or support, as well as in independent older people. In addition, the study investigated the relationship between the rate of oral *Candida* detection and clinical factors, such as medical history and oral health status.

## 2. Materials and Methods

### 2.1. Participants

This study included 31 dependent older people aged ≥70 years (nine men, 22 women; mean age, 85.1 years) who had a certified need for long-term support or nursing care, from October 2018 to October 2019. Of the 31 older people, nine required long-term support and 22 required nursing care. These individuals typically lived at home and regularly received support and care at a local day care center in Hiroshima City. In addition, this study included 27 independent older patients aged ≥70 years (six men, 21 women; mean age, 82.8 years) who visited Hiroshima University Hospital from March 2019 to December 2019. Of these 27 patients, none had a certified need for long-term support or nursing care. The study protocol was approved by the Ethical Committee of Hiroshima University, and all participants provided written informed consent.

### 2.2. Oral Examination

Oral samples were collected a few hours after participants had eaten breakfast, following tooth brushing. Therefore, most participants exhibited good oral hygiene before the oral examination and sample collection. Oral moisture was evaluated by an oral moisture-checking device (Moisture Checker Mucus^®^, Scalar Corporation, Tokyo, Japan), as previously described [7]. This device can measure the percentage of water at the surface of the oral mucosa by means of measuring the dielectric constant. The sensor of the device, protected by a disposable polyethylene cover, was applied to the measurement site at a pressure of approximately 200 g. The oral moisture level was measured at the tongue dorsum, with median values calculated from three independent measurements. A Bacterial Counter (Panasonic Healthcare Co., Ltd., Tokyo, Japan) was used to count the number of oral bacteria on the tongue surface, in accordance with the manufacturer’s instructions.

### 2.3. Oral Sample Processing and DNA Extraction

An Orcellex^®^ Brush (Rovers Medical Devices, NL, Netherlands) was used to collect samples from the tongue surface, in accordance with the method used in a prior study [8]. The tongue surface was swabbed softly 10 times, using an Orcellex^®^ Brush. Then, the brush was placed in a 1.5 mL tube containing lysis buffer (Invitrogen, Carlsbad, CA, USA). After removal of the brush, samples were centrifuged at 3000× *g* for 10 min; the supernatant was then decanted. DNA was extracted and purified using a PureLink™ Microbiome DNA Purification Kit (Invitrogen), in accordance with the manufacturer’s protocol. This kit enables purification of high-quality microbial DNA from a wide variety of sample types.

### 2.4. Detection of Candida Species by PCR

Each mixture was amplified with 1.0 µL DNA, GoTaq^®^ Green Master Mix (Promega, Madison, WI, USA), and primers. We used the following previously described PCR primer sets [9]: *Candida albicans*, 5′-TTTATCAACTTGTCACACCAGA-3′ (sense) and 5′-ATCCCGCCTTACCACTACCG-3′ (antisense); *Candida glabrata*, 5′-TTATCACACGACTCGACACT-3′ (sense) and 5′-CCCACATACTGATATGGCCTACAA-3′ (antisense); and *Candida tropicalis*, 5′-CAATCCTACCGCCAGAGGTTAT-3′ (sense) and 5′-TGGCCACTAGCAAAATAAGCGT-3′ (antisense). The PCR program consisted of initial melting at 95 °C for 5 min, followed by 35 cycles of 95 °C for 1 min, 58 °C for 1 min, and 72 °C for 1 min. After the reaction, 10 µL of PCR product was electrophoresed on a 2% agarose gel and stained with ethidium bromide. The universal 16S rRNA gene was used as control; its primer sequences were 5′-CGTTAGTAATCGTGGATCAGAATG-3′ (sense) and 5′-TGTGACGGGCGGTGTGTA-3′ (antisense).

### 2.5. Statistical Analysis

SPSS Statistics, version 24.0 (IBM Corp., Armonk, NY, USA) was used for statistical analysis. The χ^2^ test or Fisher’s exact test were used to examine significant differences between participant groups, with respect to clinical factors. Student’s *t*-test or the Mann–Whitney *U* test were used to evaluate significant differences in age, remaining teeth, degree of oral moisture, and number of oral bacteria between the two groups. Spearman’s rank correlation coefficient was used to examine the correlation between the degree of oral moisture and number of oral bacteria. *p*-values < 0.05 were considered statistically significant.

## 3. Results

### 3.1. Clinical Characteristics of Independent and Dependent Older People

Table 1 summarizes the clinical characteristics of independent and dependent older people included in this study. There were no significant differences in mean age, sex, body mass index, or medical histories between the groups. In terms of oral health status, the number of remaining teeth was significantly lower in dependent older people than in independent older people. In addition, the proportion of denture users was significantly higher among dependent older people than among independent older people. Importantly, the degree of oral moisture was significantly lower in dependent older people than in independent older people. Furthermore, the number of oral bacteria tended to be higher in dependent older people than in independent older people, but this difference was not statistically significant. To investigate the relationship between the number of oral bacteria and the degree of oral moisture, we examined the correlation between the number of bacteria and level of moisture on the tongue dorsum in the participants. We found a weak negative relationship between the number of bacteria and the level of moisture (Spearman’s rank correlation coefficient; R = −0.29, *p* = 0.01).

### 3.2. Rates of Oral Candida Detection in Dependent and Independent Older People

Table 2 summarizes the rates of oral *Candida* detection in independent and dependent older people in this study. Of the 58 participants, *C. albicans* was detected in 14 (24.1%)*, C. glabrata* was detected in 13 (22.4%), and *C. tropicalis* was detected in one (1.7%). Next, we compared the rates of *Candida* species detection between independent and dependent older people. Dependent older people exhibited significantly higher rates of *C. albicans* detection (35.5%), compared with independent older people (11.1%). Dependent older people tended to exhibit higher rates of detection of both *C. glabrata* and *C. tropicalis* (32.3% and 3.2%, respectively), compared with independent older people (11.1% and 0.0%, respectively); however, these differences were not statistically significant. In addition, both *C. albicans* and *C. glabrata* were detected concurrently in eight participants (including both independent and dependent older people). Dependent older people tended to exhibit a higher rate of concurrent detection of both *C. albicans* and *C. glabrata* (32.3%), compared with independent older people (11.1%); however, this difference was not statistically significant. Furthermore, both *C. glabrata* and *C. tropicalis* were detected concurrently in one dependent older person.

### 3.3. Clinical Characteristics of Older People Who Required Long-Term Support or Nursing Care

Table 3 summarizes the clinical characteristics of dependent older people who required long-term support or nursing care. A comparison of clinical characteristics between older people who required long-term support and older people who require nursing care revealed no significant differences in any clinical characteristics.

### 3.4. Rates of Oral Candida Detection in Older People Who Required Long-Term Support or Nursing Care

Table 4 summarizes the rates of oral *Candida* detection in dependent older people who required long-term support or nursing care. A comparison of the rates of oral *Candida* detection between older people who required long-term support and older people who required nursing care revealed that people who required nursing care tended to have higher rates of detection of *C. albicans*, *C. glabrata*, and *C. tropicalis* (36.4%, 36.4%, and 4.5%, respectively), compared with people who required long-term support (33.3%, 22.2%, and 0.0%, respectively); however, these differences were not statistically significant. In addition, both *C. albicans* and *C. glabrata* were detected concurrently in seven participants (including both people who required long-term support and people who required nursing care). People who required nursing care tended to exhibit a higher rate of concurrent detection of both *C. albicans* and *C. glabrata* (27.3%), compared with people who required long-term support (11.1%); however, this difference was not statistically significant. Furthermore, both *C. glabrata* and *C. tropicalis* were detected concurrently in one person who required nursing care.

### 3.5. Correlations Between Oral Candida Detection and Clinical Features

To investigate the association between oral *Candida* detection and clinical factors (excluding dependency status) among older people, we compared clinical characteristics between participants in whom *C. albicans* was and was not detected (i.e., *C. albicans*-positive and *C. albicans*-negative participants) (Table 5). We found no significant differences in mean age, sex, body mass index, or medical histories between the groups. In terms of oral health status, the proportion of denture users tended to be higher among *C. albicans*-positive participants (71.4%) than among *C. albicans*-negative participants (59.1%), but this difference was not statistically significant. Furthermore, there were no significant differences in mean age, sex, body mass index, or medical histories between *C. glabrata*-positive and *C. glabrata*-negative participants (Table 6). The number of remaining teeth was significantly lower in *C. glabrata*-positive participants than in *C. glabrata*-negative participants. The proportion of denture users tended to be higher among *C. albicans*-positive participants (84.6%) than among *C. glabrata*-negative participants (55.6%), but this difference was not statistically significant.

## 4. Discussion

In this study, dependent older people exhibited a significantly higher rate of detection of *C. albicans*, compared with independent older people. Thiyahuddin et al. have reported that older people living in aged care facilities showed increased rates of detection of both *C. albicans* and *C. glabrata* in the oral cavity, compared with similarly aged people living at home [10]. Therefore, dependent older people are more likely to experience oral candidiasis, compared with independent older people. In the present study, dependent older people more commonly wore dentures, compared with independent older people. The fitting surface of denture resin acts as a reservoir for microorganisms (i.e., *Candida* species) [11]. Thus, poor denture hygiene and prolonged denture use increase the risk of oral candidiasis. Denture use may be associated with high prevalence of oral *Candida* detection in dependent older people.

Histological changes in the salivary gland due to aging (i.e., decrease in number of acinar cells and increase in amount of fibrous tissue) lead to reductions in salivary flow [12]. This reduced salivary secretion attenuates immunologic and non-immunologic defenses in the oral cavity [12]. In the present study, we examined the degrees of oral moisture in dependent and independent older people of similar age. Dependent older people exhibited significantly lower levels of oral moisture, compared with independent older people. Thus, the salivary secretion capacity may be reduced in many dependent older people. Importantly, we found a significant negative correlation between the degree of oral moisture and the number of oral bacteria in older people. Sue et al. have also described a correlation between the level of moisture and the number of bacteria on the tongue surface in middle-aged and older people [8]. Therefore, we speculate that dry mouth due to aging leads to an elevated number of oral bacteria. Dependent older people are likely to exhibit an increased risk of oral candidiasis because of reduced saliva secretion and poor oral hygiene.

*C. albicans* is the predominant pathogenic yeast among *Candida* species [13]. Biofilm formation by *C. albicans* is involved in its resistance to conventional antifungal drugs [13]. The rate of oral *C. glabrata* infections has been increasing recently, and these infections have shown potent resistance to azole antifungal agents [14,15]. In addition, mixed infection by *C. albicans* and *C. glabrata* has been commonly observed in the oral cavity [16,17]. Both *C. albicans* and *C. glabrata* were frequently detected concurrently in dependent older people in the present study. Moreover, both *C. albicans* and *C. glabrata* were detected concurrently more frequently in people who required nursing care than in people who required long-term support. Both *C. glabrata* and *C. tropicalis* were detected concurrently in people who required nursing care alone. These results suggest that older people who experienced decline in activities of daily living are more susceptible to mixed oral *Candida* infection. Furthermore, mixed infection by *C. albicans* and *C. glabrata* has been shown to induce invasion of epithelium by *C. glabrata* [18]. Mixed *Candida* infection may contribute to persistent *Candida* infection in oral mucosa, which results in chronic oral candidiasis in older people. However, the exact mechanism by which *C. albicans* induces invasion of the epithelium by *C. glabrata* remains unclear.

We did not investigate the periodontal health of the older people in this study. Importantly, periodontal disease has been associated with systemic disease (e.g., diabetes, cardiovascular disease, and metabolic syndrome) [19,20]. In addition, several studies have shown that periodontitis is involved in poor oral health-related quality of life [21,22,23]. Periodontitis and tooth loss may be associated with changes in cognitive function among older adults [21]. Therefore, prevention and treatment of periodontal disease are necessary to maintain good quality of life among older people.

A previous systematic review demonstrated that patients with dementia had significantly fewer teeth and worse oral hygiene, compared with people who did not have dementia [24]. We were unable to determine the oral hygiene statuses of participants in this study. Therefore, it is unclear whether cognitive function influenced their oral hygiene status. Additional studies are needed to clarify the relationship between cognitive function and oral hygiene in older people.

## 5. Conclusions

Despite the small number of participants in this study, the results indicated that dependent older people may be more susceptible to oral *C. albicans* infection, compared with independent older people. Mixed infection by *C. albicans* and *C. glabrata* may commonly occur in dependent older people. Further research is necessary to confirm whether significant correlations are present between oral *Candida* infection and oral moisture, as well as between oral *Candida* infection and denture use, in older people.

## Figures and Tables

**Table 1:** Clinical characteristics of independent and dependent older people.

Clinical Factor (*n*)	Independent Older People (27)	Dependent Older People (31)	*p*-Value
Age (mean)	82.8 ± 5.3	85.1 ± 5.7	0.13
Sex			
Male (15)	6 (22.2%)	9 (29.0%)	0.77
Female (43)	21 (77.8%)	22 (71.0%)	
Body mass index	22.6 ± 2.8	22.3 ± 4.2	0.78
Hypertension			
No (42)	18 (66.7%)	24 (77.4%)	0.39
Yes (16)	9 (33.3%)	7 (22.6%)	
Diabetes			
No (52)	23 (85.2%)	29 (93.5%)	0.40
Yes (6)	4 (14.8%)	2 (6.5%)	
Hyperlipidemia			
No (50)	22 (81.5%)	28 (90.3%)	0.45
Yes (8)	5 (18.5%)	3 (9.7%)	
Stroke			
No (54)	25 (92.6%)	29 (93.5%)	1.0
Yes (4)	2 (7.4%)	2 (6.5%)	
Heart disease			
No (50)	22 (81.5%)	28 (90.3%)	0.45
Yes (8)	5 (18.5%)	3 (9.7%)	
Dementia			
No (53)	27 (100.0%)	26 (83.9%)	0.06
Yes (5)	0 (0.0%)	5 (16.1%)	
Remaining teeth (mean)	20.2 ± 6.3	9.1 ± 10.2	<0.001
Denture user			
Non-user (22)	16 (59.3%)	6 (19.4%)	0.003
User (36)	11 (40.7%)	25 (80.6%)	
Degree of oral moisture	28.7 ± 1.7	26.6 ± 2.9	0.003
Number of oral bacteria (1.0 × 10^6^ [CFU]/mL)	9.4 ± 10.2	13.2 ± 12.3	0.26

*p*-values < 0.05 were considered statistically significant.

**Table 2:** Rates of oral *Candida* in independent and dependent older people.

Clinical Factor (*n*)	Independent Older People (27)	Dependent Older People (31)	*p*-Value
*C. albicans*			
Negative (44)	24 (88.9%)	20 (64.5%)	0.04
Positive (14)	3 (11.1%)	11 (35.5%)	
*C. glabrata*			
Negative (45)	24 (88.9%)	21 (67.7%)	0.07
Positive (13)	3 (11.1%)	10 (32.3%)	
*C. tropicalis*			
Negative (57)	27 (100.0%)	30 (96.8%)	1.0
Positive (1)	0 (0.0%)	1 (3.2%)	
*C. albicans/C. glabrata*			
Negative (50)	25 (92.6%)	25 (80.6%)	0.26
Positive (8)	2 (7.4%)	6 (19.4%)	
*C. glabrata/C. tropicalis*			
Negative (57)	27 (100.0%)	30 (96.8%)	1.0
Positive (1)	0 (0.0%)	1 (3.2%)	

*p*-values < 0.05 were considered statistically significant.

**Table 3:** Clinical characteristics of older people who required long-term support or nursing care.

Clinical Factor (*n*)	People Requiring Support (9)	People Requiring Nursing Care (22)	*p*-Value
Age (mean)	86.3 ± 4.5	84.6 ± 6.2	0.59
Sex			
Male (9)	3 (33.3%)	6 (27.3%)	1.00
Female (22)	6 (66.7%)	16 (72.7%)	
Body mass index	21.8 ± 3.8	22.5 ± 4.5	0.54
Hypertension			
No (24)	7 (77.8%)	17 (77.3%)	1.0
Yes (7)	2 (22.2%)	5 (22.7%)	
Diabetes			
No (29)	8 (88.9%)	21 (95.5%)	0.50
Yes (2)	1 (11.1%)	1 (4.5%)	
Hyperlipidemia			
No (28)	8 (88.9%)	20 (90.9%)	1.0
Yes (3)	1 (11.1%)	2 (9.1%)	
Stroke			
No (29)	9 (100.0%)	20 (90.9%)	1.0
Yes (2)	0 (0.0%)	2 (9.1%)	
Heart disease			
No (28)	7 (77.8%)	21 (95.5%)	0.20
Yes (3)	2 (22.2%)	1 (4.5%)	
Dementia			
No (26)	9 (100.0%)	17 (77.3%)	0.29
Yes (5)	0 (0.0%)	5 (22.7%)	
Remaining teeth (mean)	15.9 ± 0.02	16.1 ± 0.02	0.92
Denture user			
Non-user (6)	2 (22.2%)	4 (18.2%)	1.0
User (25)	7 (77.8%)	18 (81.8%)	
Degree of oral moisture	25.1 ± 3.5	27.3 ± 2.4	0.14
Number of oral bacteria (1.0 × 10^6^ [CFU]/mL)	12.8 ± 7.8	13.4 ± 13.9	0.54

*p*-values < 0.05 were considered statistically significant.

**Table 4:** Rates of oral *Candida* in older people who required long-term support or nursing care.

Clinical Factor (*n*)	People Requiring Support (9)	People Requiring Nursing Care (22)	*p*-Value
*C. albicans*			
Negative (20)	6 (66.7%)	14 (63.6%)	1.0
Positive (11)	3 (33.3%)	8 (36.4%)	
*C. glabrata*			
Negative (21)	7 (77.8%)	14 (63.6%)	0.68
Positive (10)	2 (22.2%)	8 (36.4%)	
*C. tropicalis*			
Negative (30)	9 (100.0%)	21 (95.5%)	1.0
Positive (1)	0 (0.0%)	1 (4.5%)	
*C. albicans/C. glabrata*			
Negative (24)	8 (88.9%)	16 (72.7%)	0.64
Positive (7)	1 (11.1%)	6 (27.3%)	
*C. glabrata/C. tropicalis*			
Negative (30)	9 (100.0%)	21 (95.5%)	1.0
Positive (1)	0 (0.0%)	1 (4.5%)	

*p*-values < 0.05 were considered statistically significant.

**Table 5:** Correlation between *C. albicans* detection and clinical factors among independent and dependent older people.

Clinical Factor (*n*)	*C. albicans*		*p*-Value
	(-) (*n* = 44)	(+) (*n* = 14)	
Age (mean)	83.6 ± 5.8	85.4 ± 4.9	0.28
Sex			
Male (15)	11 (25.0%)	4 (28.6%)	0.77
Female (43)	33 (75.0%)	10 (71.4%)	
Body mass index	22.3 ± 3.6	23.0 ± 4.2	0.29
Hypertension			
No (42)	29 (65.9%)	13 (92.9%)	0.09
Yes (16)	15 (34.1%)	1 (7.1%)	
Diabetes			
No (52)	40 (90.9%)	12 (85.7%)	0.62
Yes (6)	4 (9.1%)	2 (14.3%)	
Hyperlipidemia			
No (50)	38 (86.4%)	12 (85.7%)	1.0
Yes (8)	6 (13.6%)	2 (14.3%)	
Stroke			
No (54)	40 (90.9%)	14 (100%)	0.56
Yes (4)	4 (9.1%)	0 (0%)	
Heart disease			
No (50)	38 (86.4%)	12 (85.7%)	1.0
Yes (8)	6 (13.6%)	2 (14.3%)	
Dementia			
No (53)	40 (90.9%)	13 (92.9%)	1.0
Yes (5)	4 (9.1%)	1 (7.1%)	
Remaining teeth (mean)	15.0 ± 10.0	12.0 ± 10.7	0.36
Denture user			
Non-user (22)	18 (40.9%)	4 (28.6%)	0.53
User (36)	26 (59.1%)	10 (71.4%)	
Degree of oral moisture	27.8 ± 2.5	26.9 ± 2.9	0.26
Number of oral bacteria (1.0 × 10^6^ [CFU]/mL)	10.5 ± 10.8	14.3 ± 13.4	0.35

*p*-values < 0.05 were considered statistically significant.

**Table 6:** Correlation between *C. glabrata* detection and clinical factors among independent and dependent older people.

Clinical Factor (*n*)	*C. glabrata*		*p*-Value
	(-) (*n* = 45)	(+) (*n* = 13)	
Age (mean)	83.6 ± 6.0	85.5 ± 3.4	0.29
Sex			
Male (15)	12 (26.7%)	3 (23.1%)	1.0
Female (43)	33 (73.3%)	10 (76.9%)	
Body mass index	22.3 ± 3.6	23.0 ± 4.2	0.53
Hypertension			
No (42)	30 (66.7%)	12 (92.3%)	0.09
Yes (16)	15 (33.3%)	1 (7.7%)	
Diabetes			
No (52)	40 (88.9%)	12 (92.3%)	1.0
Yes (6)	5 (11.1%)	1 (7.7%)	
Hyperlipidemia			
No (50)	38 (84.4%)	12 (92.3%)	0.67
Yes (8)	7 (15.6%)	1 (7.7%)	
Stroke			
No (54)	41 (91.1%)	13 (100%)	0.57
Yes (4)	4 (8.9%)	0 (0%)	
Heart disease			
No (50)	40 (88.9%)	10 (76.9%)	0.36
Yes (8)	5 (11.1%)	3 (23.1%)	
Dementia			
No (53)	42 (93.3%)	11 (84.6%)	0.31
Yes (5)	3 (6.7%)	2 (15.4%)	
Remaining teeth (mean)	16.5 ± 9.6	6.7 ± 8.0	0.01
Denture user			
Non-user (22)	20 (44.4%)	2 (15.4%)	0.10
User (36)	25 (55.6%)	11 (84.6%)	
Degree of oral moisture	27.7 ± 2.7	27.3 ± 2.1	0.63
Number of oral bacteria (1.0 × 10^6^ [CFU]/mL)	10.6 ± 11.4	14.2 ± 11.7	0.24

*p*-values < 0.05 were considered statistically significant.

## Data Availability

Data relevant to this study are included in the article.
